# The Role of Deubiquitinating Enzymes in Hematopoiesis and Hematological Malignancies

**DOI:** 10.3390/cancers12051103

**Published:** 2020-04-28

**Authors:** Neha Sarodaya, Janardhan Karapurkar, Kye-Seong Kim, Seok-Ho Hong, Suresh Ramakrishna

**Affiliations:** 1 Graduate School of Biomedical Science and Engineering, Hanyang University, Seoul 04763, Korea; neyha19@gmail.com (N.S.); kalpesh25021992@gmail.com (J.K.); ks66kim@hanyang.ac.kr (K.-S.K.); 2 College of Medicine, Hanyang University, Seoul 04763, Korea; 3 Department of Internal Medicine, School of Medicine, Kangwon National University, Chuncheon 24341, Korea

**Keywords:** DUBs, HSCs, myeloid, erythroid, leukemia, lymphoma, cell differentiation

## Abstract

Hematopoietic stem cells (HSCs) are responsible for the production of blood cells throughout the human lifespan. Single HSCs can give rise to at least eight distinct blood-cell lineages. Together, hematopoiesis, erythropoiesis, and angiogenesis coordinate several biological processes, i.e., cellular interactions during development and proliferation, guided migration, lineage programming, and reprogramming by transcription factors. Any dysregulation of these processes can result in hematological disorders and/or malignancies. Several studies of the molecular mechanisms governing HSC maintenance have demonstrated that protein regulation by the ubiquitin proteasomal pathway is crucial for normal HSC function. Recent studies have shown that reversal of ubiquitination by deubiquitinating enzymes (DUBs) plays an equally important role in hematopoiesis; however, information regarding the biological function of DUBs is limited. In this review, we focus on recent discoveries about the physiological roles of DUBs in hematopoiesis, erythropoiesis, and angiogenesis and discuss the DUBs associated with common hematological disorders and malignancies, which are potential therapeutic drug targets.

## 1. Introduction

Blood consists of red blood cells (RBCs), megakaryocytes, myeloid cells (monocytes/macrophages and neutrophils) and lymphocytes. All these components are produced by rare cells in the bone marrow called hematopoietic stem cells (HSCs) through a process known as hematopoiesis [1]. The properties of self-renewal and differentiation into various progenitor cells allow HSCs to reconstitute the entire blood system. The progenitor cells further mature into lineage-specific precursors by specific pathways. The hematopoietic system originates in two sites: the yolk sac (primitive, generating nucleated erythrocytes) and the aorta-gonad mesonephros region (definitive, generating HSCs that give rise to all blood lineages) [2,3]. 

Erythropoiesis is the differentiation of multipotent hematopoietic stem cells to unipotent stem cells that are primitive erythroid progenitors [4]. Primitive or embryonic erythropoiesis is the maturation of erythroid progenitors from yolk sacs in the fetal liver. After birth, definitive erythropoiesis is initiated in bone marrow in successive waves of erythroid progenitor-cell maturation, including generation of burst-forming unit erythroids, colony-forming unit erythroids (CFU-Es), proerythroblasts, and basophilic, polychromatic, and orthochromatic erythroblasts derived from HSCs [5,6]. A CFU-E undergoes many substantial changes, such as chromatin condensation and enucleation, and gives rise to erythrocytes or RBCs. Erythrocyte production is regulated at each stage of development through transcription factors, post-translational modification of histones, and the interplay between the cell cycle and RBC differentiation [7,8].

Angiogenesis is the formation of blood vessels from the existing vascular system. Mesodermal stem cells are the source of HSCs and angioblasts in the embryo. Mesodermal cells in the embryo form aggregates of endothelial precursor cells or angioblasts called blood islands [9,10]. These blood islands fuse to form hierarchical networks of arteries, capillaries, and veins, whereas HSCs mature to form the components of blood [9]. The complex network of blood vessels produced by angiogenesis carries oxygenated RBCs throughout the body, along with other blood cells, macromolecules, gases, and fluids [9,10]. The circulation of blood to all tissues enables diffusion and exchange of nutrients and metabolites within the large and complex bodies of vertebrates. Angiogenesis is essential for both normal growth and development and for the growth of tumors and metastasis [11], and identifying the regulators of angiogenesis is of potential therapeutic benefit.

HSC differentiation is a tightly controlled process, and recent studies suggest that post-translational modification of protein substrates plays an essential role in its regulation. Many studies have established the roles of ubiquitination and deubiquitination in regulating a wide range of transcription factors, signal transduction pathways, and niche factors [12]. Normal hematopoiesis requires tight regulation of the expression of lineage-specific genes and of epigenetic and post-translational modifications, and any perturbations in this process may result in myeloid or lymphoid disorders or malignancies [1]. 

## 2. Ubiquitination and Deubiquitination

**Figure 1:**
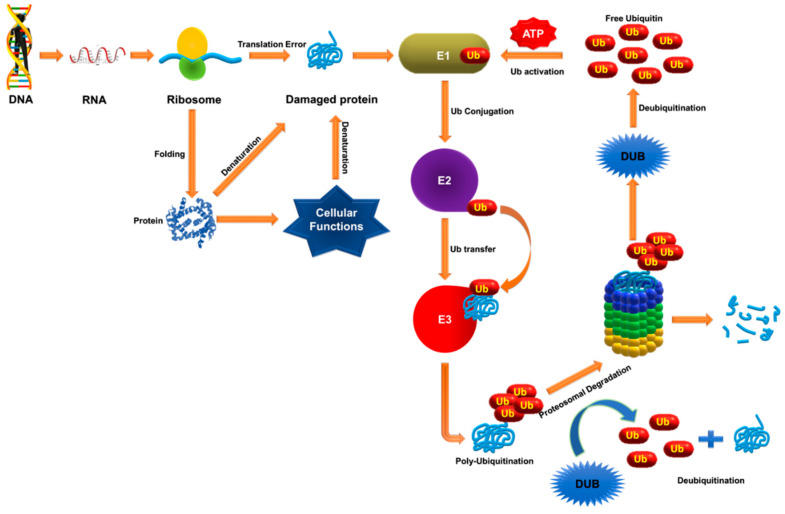
Protein quality control and degradation by the ubiquitin proteasome system (UPS). Nascent polypeptides and misfolded proteins that arise from translational errors are the targets of the UPS, and involves balancing myriad intracellular protein levels. The UPS consists mainly of ubiquitination of the target protein by E3 ligases and degradation of ubiquitinated proteins by the proteasome. Ubiquitination is tagging of a target protein. This cascade of enzyme reactions is catalyzed by the sequential activity of three enzymes: E1 (ubiquitin-activating enzyme), E2 (ubiquitin-conjugating enzyme) and E3 (ubiquitin ligase). This process is counterbalanced by Ub proteases belonging to either metalloprotease or cysteine protease. These DUBs cleave the ubiquitin molecule from the substrate protein, maintaining the pool of mono-Ub which is supplied for the ubiquitination of misfolded proteins. Any disturbance in the equilibrium between ubiquitination and deubiquitination can induce proteotoxicity.

Ubiquitination is the process whereby an ubiquitin, a 76-residue polypeptide, is covalently attached to a protein substrate. Conjugation of ubiquitin to its substrate protein is carried out by large enzymatic complexes: ubiquitin-activating enzymes (E1), ubiquitin-conjugating enzymes (E2), and ubiquitin ligases (E3). The ubiquitin molecule is activated first by E1 enzymes in an ATP-dependent manner, forming a thiol ester bond with the C-terminus of ubiquitin, which is then transferred to E2 through a thioester-linked E2-ubiquitin intermediate. Next, the E2 and E3 enzymes position the target protein substrate and transfer this activated ubiquitin to its lysine residue (Figure 1) [13]. Ubiquitin has certain specific lysine residues for ubiquitin conjugation, which modify the protein in a specific way. Protein modification by attachment of a single ubiquitin molecule is termed monoubiquitination, while conjugation of single or multiple lysine residues by a chain of ubiquitin oligomers is polyubiquitination. Monoubiquitination of a protein is associated with DNA repair, vesicle sorting, signal transduction and receptor endocytosis, and polyubiquitination is associated with protein degradation by the 26S proteasome. The addition of ubiquitin is reversible. Deubiquitinating enzymes (DUBs) are proteases that remove ubiquitin from ubiquitin-protein conjugates prior to proteolysis (Figure 1). DUBs are an important class of enzymes, as they can negatively regulate protein degradation and help balance the pool of circulating free ubiquitin [14,15,16].

DUBs belong to the human family of proteases, which catalyze the removal of ubiquitin from substrate proteins and play a vital role in ubiquitin recycling, editing, and maturation [17,18]. DUBs also help modulate cellular pathways, including gene expression [19], apoptosis [20], cell cycling [21], cellular reprogramming [22], oogenesis [23], spermatogenesis [24], endosomal trafficking and endocytosis [25], and proteostasis [26]. Approximately 100 different types of DUBs are expressed by the human genome, and they can be subdivided into six different subfamilies according to sequence conservation in the catalytic domain: (1) ubiquitin-specific proteases (USP), (2) ubiquitin C-terminal hydrolases, (3) OTU-domain-containing ubiquitin aldehyde-binding protein 1 (OTUB1), (4) ataxin-3/Machado-Joseph domain proteases, (5) JAB1/Pad/MOV34/MPN-domain containing metallo- enzymes [27], and (6) monocyte chemotactic protein-induced proteases [28].

Rehman et al. reported the discovery of a new family of DUBs called MINDY (motif interacting with Ub-containing novel DUB family), which shows high selectivity for cleavage of K48-linked polyubiquitin chains [29]. In addition to these families, M48^USP^, a novel class of protease that functions as a DUB, has also been documented [26]. However, the precise functions and substrates for most DUBs have yet to be elucidated.

A growing body of evidence indicates that DUBs play important roles in the differentiation of hematopoietic stem cells to all blood-cell lineages, including lymphoid-lineage T and B cells, and myeloid-lineage neutrophils, eosinophils, basophils, monocytes, macrophages, megakaryocytes, and platelets [30,31]. In this review, we discuss the evolving understanding of the role of DUBs in hematopoiesis, erythropoiesis, and angiogenesis, and the diseases associated with these processes. 

## 3. Importance of DUBs in Hematopoiesis

### 3.1. DUB-1, DUB-2A, and DUB-3

Recently, a hematopoietic-specific, cytokine-inducible, growth-regulatory subfamily of DUBs was identified. Within this family, DUB-1, an immediate-early gene, is induced by interleukin (IL)-3 and IL-5 in the murine hematopoietic progenitor cell line Ba/F3. DUB-1 regulates ubiquitin-dependent proteolysis, which arrests the cell cycle in the S phase. DUB-1 is specifically expressed in hematopoietic cells and may play a role in growth suppression [32]. Another immediate-early gene, DUB-2, is induced by IL-2 in the cytokine-dependent murine hematopoietic progenitor cell line CTLL. DUB-2 inhibits apoptosis after cytokine withdrawal [33] and prolonged STAT5 phosphorylation [34]. DUB-2 influences STAT5 activation, which is important for the expression of various oncogenes [34]. DUB-2A, which is similar to DUB-2, is induced by IL-2 and regulates intracellular cytokine-induced growth proteins such as CBL, E3 ubiquitin- protein ligase [35] and cytokine-induced STAT inhibitor protein [36,37]. The proteins involved in cytokine-induced signaling pathways are regulated by ubiquitin-dependent proteolysis in hematopoietic cells. This regulation involves two important processes: ubiquitin-dependent internalization and turnover of cytokine receptors [38,39] and STAT1 degradation [40].

DUB-3 or USP17, induced by IL-4 and IL-6, plays an important role in blocking cell proliferation and inducing apoptosis in hematopoietic cells [41,42]. DUB-1 and DUB-2 are specifically expressed in either B or T cells, respectively, whereas DUB-3 is found in numerous hematopoietic tumors [34,42]. These DUBs are induced by hematopoietic cytokines to initiate a cytokine-specific growth response; thereafter, DUB-1 and DUB-2 degrade as the cytokine response is downregulated [32,33,43]. Activation of specific DUBs by certain cytokines may therefore regulate various cellular signaling events promoting cell growth or differentiation.

### 3.2. MYSM1

MYSM1, originally identified as a histone H2A deubiquitinase, plays an important role in activating several genes regulated by androgen receptors in prostate cancer cells [44]. Transcription factors are key determinants of the complex orchestration of hematopoiesis, but little is known about the underlying mechanisms by which these transcription factors are regulated [45]. Transcription of a gene is critically regulated by epigenetic histone modifications. EBF1 is one of the master transcription factors for B-cell lineage commitment and development, and its deficiency leads to a blockage in early pro-B-cell development [46]. EBF1 plays an important role in the activation of many genes, including Pax5, Foxo1, Cd79a, Cd79b, Igll1, and others that are important for B-cell development [47,48]. MYSM1 plays multiple roles in the hematopoietic system and regulates the transcription of Ebf1, as well as other genes essential in B-cell development [49]. MYSM1 also plays an essential role in B-cell development, hematopoietic development and lymphocyte generation in the bone marrow of both humans [50] and mice [45]. Several studies have found that MYSM1 deficiency or mutation can lead to defects during the development and function of HSCs, B cells, natural killer (NK) cells and dendritic cells, and it also results in the development of lymphopenia, anemia, and thrombocytopenia, and low B-cell and NK-cell counts in both mice and humans [45,51,52,53,54,55]. 

### 3.3. USP3

USP3 regulates the ubiquitin-dependent DNA damage response (DDR) to double-stranded DNA breaks [56]. In USP3-knockout HSCs, defective ubiquitination has been shown to decrease HSC function, and increase cumulative DNA damage and hypersensitivity to ionizing radiation [57]. Due to the loss of USP3, HSCs in USP3 knockout mice are under chronic genotoxic stress, causing shortened lifespan and associated functional declines in the hematopoietic stem and progenitor compartment. USP3 appears to protect HSCs against DNA damage by regulating DDR signaling [58].

### 3.4. USP16

Another H2A deubiquitinase, USP16, regulates many genes involved in hematopoiesis [59]. For example, USP16 regulates cell cycling during hematopoiesis through the polycomb repressive complex 1 (PRC1), a major H2A ubiquitin ligase. Some studies have suggested deleting USP16 can affect HSC lineage commitments by reducing the number of mature progenitor cells [60]. USP16 also plays a role in the expression of genes involved in HSC differentiation, chromosome organization immune response and hematopoietic organ development [60,61] and it regulates the transition between HSCs with long-term regeneration capacities (long-term HSCs) to those with short-term regeneration capacities during fetal hematopoiesis [62].

### 3.5. USP1 and USP10

During normal development, HSCs are prone to apoptosis due to fluctuations in cytokine availability as they move from the fetal liver (FL) to bone marrow (BM). USP10 inhibits apoptosis by two distinct mechanisms: deubiquitinase-independent/ROS-dependent and deubiquitinase-dependent/ROS-independent. The latter mechanism inhibits cytokine-deprivation-induced apoptosis of hematopoietic stem and progenitor cells, including long-term HSCs in the FL. BM failure with pancytopenia and anemia due to pronounced reduction of HSCs and progenitor cells is also observed in USP10-knockout mice [31]. Another DUB involved in bone marrow failure is USP1, which is associated with a chromosome instability syndrome called Fanconi anemia (FA). USP1 regulates the FA pathway by deubiquitinating FANCD2. USP1 inhibition leads to accumulation of monoubiquitinated FANCD2 and protects cells against certain types of DNA damage [63,64].

### 3.6. USP15

A recent study by Van Den Berk et al. in mouse hematopoietic progenitor cells revealed that USP15, along with USP4 and USP11, are essential for hematopoietic stem and progenitor cell maintenance. USP15 knockout mice showed reduced HSC levels and a stable differentiated progenitor pool, suggesting a role in HSC homeostasis. Also, because USP15 is highly expressed in primary blood–derived tumors, chronic myeloid leukemia (CML) and acute myeloid leukemia (AML) patients, it can serve as a target for cancer therapy. USP15 inhibition leads to chromosomal aberration, making USP15 a potential target against leukemia [65]. Tumor necrosis factor (TNF) receptor–associated factor (TRAF)-interacting protein with a forkhead-associated domain (TIFA) and its structural homolog, TIFAB, are involved in various innate immune signaling pathways associated with hematopoietic malignancies. TIFAB forms a complex with USP15, increasing the rate of USP15 deubiquitination and regulating p53 signaling in malignant hematopoietic cells [66].

### 3.7. Other DUBs

BRCA1-associated protein 1 (BAP1) is an essential component of the polycomb repressive deubiquitinase complex (PR-DUB), deubiquitinating monoubiquitinated histone H2A at lysine 119 (H2AK119ub) in a modification catalyzed by the PRC1. The mammalian PR-DUB complex contains ASXL family proteins, which are required for deubiquitinating activities and often mutated in myelodysplastic syndrome [67]. Another DUB, USP42, is expressed in bone marrow and is associated with RUNX1 expression (a key regulator of hematopoiesis) in AML [68]. Other histone deubiquitinases such as USP16, MYSM1, and USP21 are essential for hematopoiesis and hematopoietic stem cell function [49].

## 4. Importance of DUBs in Erythropoiesis and Angiogenesis

Erythropoiesis involves reorganization of a complex cellular compartment in support of the differentiation and maturation of RBCs. This reorganization is regulated in part by programmed protein degradation [69]. During erythropoiesis, protein ubiquitination and deubiquitination guide the removal of proteins and organelles through proteasomes or lysosomes. However, the role of DUBs in erythropoiesis requires further investigation.

Among the USP subfamily, USP50 is considered catalytically inactive due to the unavailability of one of the canonical residues required for catalysis [70]. USP50 is upregulated during the terminal stages of erythropoiesis, and can be involved in cell-cycle arrest [8]. A DNA binding protein, Ku70, is expressed in CD34^+^ hematopoietic progenitor cells. USP50 regulates Ku70-mediated deubiquitination of myeloid leukemia-cell differentiation protein, which plays an important role in regulating apoptosis in the later stages of erythropoiesis [8,71]. To understand the exact molecular mechanism behind USP50-mediated destabilization of Ku-70, USP-50 knockout mice may provide some useful insights [8]. According to previous studies, a Mysm1-knockout mouse line had defects in lymphopoiesis and erythropoiesis, which play important roles in the maintenance of lymphocyte and erythrocyte differentiation. Importantly, MYSM1 was also linked to the generation of reactive oxygen species, DNA damage and impaired survival of Mysm1-knockout hematopoietic progenitors. The studies also found that Mysm1-knockout mouse lines mimic the symptoms of human aplastic anemia, lymphopenia, and thrombocytosis. MYSM1 therefore plays an essential role in HSC function; however the association of MYSM1 with these human disorders requires further investigation [72]. USP7 reportedly ubiquitinates and stabilizes the erythroid transcription factor GATA. Originally identified as a potential anti-cancer target in a genome-wide RNA interference (RNAi) screen of catalytically active USPs, USP7 has recently attracted attention as a potential therapeutic target. According to a recent study, USP7 expression was significantly upregulated during erythropoiesis, and associated with delayed terminal erythroid differentiation, inhibition of hemoglobin expression and cell proliferation, and induction of apoptosis [73].

Angiogenesis refers to the migration, growth, and differentiation of vascular endothelial cells to form new capillary blood vessels. This process is tightly regulated by a range of angiogenic factors and inhibitors, the most important of which is vascular endothelial growth factor. Protein ubiquitination regulates virtually every aspect of the angiogenesis signaling pathway. Both the canonical and non-canonical Wnt signaling pathways are important in angiogenesis. The key component of Wnt signaling is the disheveled protein, which helps cleave linear ubiquitin linkages [12]. Linear ubiquitination linked via Ub Met-1 (M1) chains regulates nuclear factor-κB (NF-κB)-dependent inflammation and adaptive immunity. The linear ubiquitin chain assembly complex (LUBAC) consists of HOIL-1/1l, HOIP and SHARPIN proteins, which together produce M1-linked chains. Certain DUBs, such as CYLD and OTULIN (also known as Gumby or *Fam105b*), are known to negatively regulate LUBAC. OTULIN binds to LUBAC and decreases linear ubiquitination, thereby activating NF-κB-dependent transcription [12,74]. OTULIN and CYLD can therefore be used to develop anti-angiogenic therapy. 

Studies have reported that the angiogenesis-associated NF-κB signaling pathway is negatively regulated by A20 through TNFα-mediated inhibition. TNF-α inhibition reduced angiogenesis in rheumatoid arthritis patients and A20 is considered to be pro-angiogenic [75]. RNAi-induced downregulation of A20 expression in primary human umbilical vein endothelial cells reportedly resulted in impaired endothelium tube formation. The exact mechanism by which A20 regulates angiogenesis is currently unknown, but several reports discuss the role of A20 in cell differentiation, proliferation, and migration [76,77]. Sprouting is a fundamental process involved in angiogenesis. The sprouting behavior of endothelial cells is controlled in part by the Notch receptor protein. The deletion of USP10 upregulates NOTCH1, promoting angiogenic sprouting [78]. Monocyte chemotactic protein–induced protein 1 (MCPIP1) reportedly acts as a DUB that can remove the ubiquitin from proteins [79]. Hypoxia-inducible factor-1 α, which promotes angiogenesis, is deubiquitinated and stabilized by MCPIP1 [80,81]. Preclinical and clinical studies have suggested that angiogenic inhibitors can be useful against drug-resistant tumors as they do not kill cancer cells directly but attenuate angiogenesis. DUB inhibitors may therefore prove to be a novel target in anti-angiogenic therapy against drug-resistant cancers and other angiogenic disorders. 

## 5. DUBs in Hematological Malignancies

UPS, along with DUBs, are involved in catalyzing the destruction of many protein substrates in the pathogenesis of several types of cancers. Dysregulation of this proteolysis-regulating machinery can result in uncontrolled cell proliferation, accumulation of harmful proteins, and genetic instability, leading ultimately to malignancy [82,83]. Several DUBs that are dysregulated by various processes in hematological malignancies have been identified. These perturbations in the turnover of regulatory proteins lead to disruptions of cellular homeostasis and disturb the balance of various signaling pathways contributing to the multistep process of carcinogenesis [84]. However, there is limited evidence of direct links between neoplastic transformation and dysregulated deubiquitination [85]. The potential for therapeutic targeting of the DUBs that are linked to various hematological malignancies is described in the preceding section.

### 5.1. USP1

The helix loop helix transcriptional regulatory protein family consists of four members, inhibitor of DNA binding 1 through 4 (ID1–ID4). Among these, ID1 is overexpressed in several cancers, such as colon, pancreatic, breast and brain cancers [86,87,88,89]. ID1 is more highly expressed in multiple myeloma (MM) and AML samples compared with normal cells. It also promotes stem-like characteristics in normal and malignant tissues [90]. ID1 is involved in the cellular differentiation pathway and is also a downstream target of USP1 [91]. USP1 physically associates and deubiquitinates the FA protein FANCD2 when cells exit the S phase or recommence cycling after a DNA-damaging insult and may play a critical role in DNA repair [63]. USP1 inhibitor SJB (SJB3-019A) initiates apoptosis in MM cells and inhibits DNA repair by impeding the FA repair pathway [92]. Another known inhibitor of USP1, pimozide, is approved by the U.S. Food and Drug Administration for treating Tourette syndrome. Pimozide also supports degradation of ID1 and reduced viability of leukemic cells [91]. These small-molecule inhibitors therefore promote degradation of ID1, a prime therapeutic target, and require further preclinical assessment to determine their efficacy for cancer therapy.

### 5.2. A20

A20 is a protein that protects against TNF-induced cytotoxicity and has been shown to have both E3 ligase and deubiquitinase activity. Inactivation or deletion of A20 is associated with several types of hematological malignancies [93]. Previous studies have shown that activation of the NF-κB pathway plays an important role in the pathogenesis of various hematological malignancies. Several studies have demonstrated that NF-κB activation is negatively regulated by A20 through interactions with pro-inflammatory proximal signaling proteins such as TRAF1, TRAF2, TRAF6, RIPK1, RIPK2, NEMO, and MALT1, which are necessary for the activation of the NF-κB signaling cascade [94,95]. Owing to the potential of the NF-κB suppressor function of A20, and given the link between tumorigenesis and NF-κB mediated chronic inflammation, the discovery of inactivation of A20 by genomic deletion, somatic mutation, or epigenetic hypermethylation in several types of hematological malignancies was not surprising [96,97,98]. It has been observed that A20 is frequently inactivated in B-cell lymphomas accompanied by upregulation of NF-κB activation. Reintroduction of wild-type A20 alleles in these lymphoma-derived cell lines resulted in apoptosis and suppression of cell growth, along with downregulation of the NF-κB pathway [99,100].

Knockdown or inactivation of A20 resulted in cell proliferation in acute T-cell lymphocytic leukemia [97], mucosa-associated lymphoid tissue (MALT) lymphoma [101,102], marginal zone lymphoma [103,104], primary mediastinal B-cell lymphoma, and classic Hodgkin’s lymphoma [105,106], suggesting that A20 has tumor-suppressor abilities. Inactivation of A20 by biallelic mutation was also detected in cell lines derived from patients with diffuse and large B-cell lymphoma, MALT lymphoma, Hodgkin’s lymphoma, or marginal zone lymphoma [98,100,103,107] further confirming the tumor-suppressor activity associated with A20. Several studies concluded that A20 functions through various mechanisms such as regulation of proximal signaling proteins; early degradation of several oncogenes associated with hyperactivated signaling cascades, and blocking TNFα-induced NF-κB signaling cascades [95].

Inactivation mutation in the A20 gene provides a survival advantage for tumor cells and identifying such mutations in lymphomas that can restore tumor-suppressive function will provide insight on regions that are crucial for tumor suppression. Researchers have identified diverse sets of mutations in different regions of A20 gene in various hematological malignancies [95], reflecting the complexity of the tumor-suppressive function of A20. Although reports show that tumor-suppressor activity in A20 is multifaceted and a result of several mechanisms working in concert, the exact molecular mechanism or mode of action of A20 has not been completely elucidated. However, its involvement in the regulation of ubiquitin-dependent signaling and the link between A20 inactivation and tumorigenesis indicates that A20 is a potential therapeutic target in various hematological malignancies. Alternatively, as upregulation of NF-κB signaling is associated with A20 inactivation in various hematological malignancies, selectively targeting pro-inflammatory receptors with inhibitors may offer therapeutic benefits. Antagonizing drugs such as rituximab, which targets CD20 [108,109]; adalimumab; infliximab; etanercept, which bind to TNF signaling receptors [110]; or caspase 3/7 activation and induction of lactate dehydrogenase release by ectinascidin 743, emetinec, and chromomycin A3 [111] may offer therapeutic benefits in hematological malignancies with A20 inactivation.

### 5.3. USP7

USP7 was initially identified as ICP0 (herpes simplex virus protein)-stabilizing protein [112]. Several studies have reported that USP7 mediates stabilization of ICP0 enzyme–inducing proteasome-dependent degradation of a number of proteins, including p53 and promyelocytic leukemia protein by protecting it from auto-ubiquitination [113]. MDM2, the murine double-minute oncogene (HDM2-human orthologue), is a substrate for USP7 which negatively regulates the tumor-suppressor protein p53. Under normal conditions, USP7 stabilizes intracellular MDM2 concentrations, which in turn drive steady ubiquitination of p53, which targets it for proteasome-mediated degradation [114]. In MM, reduction in p53 expression occurs at the later stages of cancer, along with overexpression of USP7. Studies have shown that inhibition of USP7 causes auto-ubiquitination and degradation of MDM2, resulting in p53 stabilization inducing apoptosis via G1 phase arrest [115]. The UPS appears to play an essential role in tumorigenesis, and preclinical and clinical studies have helped develop proteasome inhibitor bortezomib as a target against MM. However, recent studies have demonstrated possible off-target toxicity, development of resistance toward bortezomib, and its limited application in p53-deficient MM cells [116,117]. Given the link between upregulation of USP7 and tumor aggressiveness in MM, an alternate therapeutic approach of using USP7 inhibitors would represent a major advance. Downregulation of USP7 by P5091 (a USP7 inhibitor) in MM cell lines, an MM xenograft model, and patient-derived tumor cells can create a potent and specific inhibitor that enhances degradation of HDM2, as well as upregulation of p53 and p21 expression, resulting in cell cytotoxicity [118]. USP7 regulates p53 activity by deubiquitinating and stabilizing it, and overexpression of USP7 induces p53-dependent apoptosis. The N-terminal domain of USP7 is involved in p53-USP7 interactions, and also contains a TRAF domain and an EBNA1 binding domain. Human TRAF regulates lymphocyte survival, while EBNA1 is a viral onco-protein responsible for the immortalization of cells and development of B-cells lymphomas [119]. Chronic lymphocytic leukemia (CLL) is underscored by genetic aberrations at a particular locus in ATM and p53 genes, which are associated with DDR. These genes play critical roles in DDR-mediated tumor suppression. Defects in these genes facilitate accumulation and persistence of genetic mutations, increasing genomic instability, which ultimately results in tumorigenesis as well as therapeutic drug resistance. 

Recent studies have reported that USP7 modulates the stability of RAD18, a DNA damage-responsive E3 ubiquitin ligase, which in turn regulates p53 expression in CLL. In CLL models and patient-derived tumor samples, ATM-p53 mediated DDR is inactivated, while expression of USP7 was upregulated, a relationship which correlates the findings of a study of an MM model showing similar overexpression of USP7. Inhibition of USP7 by the chemotherapeutic inhibitor HBX19818 and siRNA-mediated downregulation of USP7 resulted in significant increases in tumor-cell apoptosis and disruption in homologous recombination repair due to genotoxicity [120]. Preclinical studies have shown that USP7 small-molecule inhibitors such as P22077, P5091, GNE-6640, GNE-6776, and HBX19818 are well tolerated, induce efficient tumor-cell cytotoxicity, and selectively inhibit target molecules in in vitro and in vivo conditions [118,121]. The effect of USP7 on the turnover of p53 and DDR-associated proteins, along with the efficacy of USP7 inhibitors on hematological malignancies, provides a proof of concept for the evaluation of USP7 as a potential pharmacological target in hematological tumors [120].

### 5.4. USP9X

USP9X is a substrate-specific DUB with a highly conserved sequence in *Drosophila* and humans. Overexpression of USP9X is associated with poor prognosis in various cases of hematological malignancies, such as CML, B-cell malignancies, and MM. Studies have shown that MM patients overexpressing USP9X are at higher risk of death and are associated with a poor prognosis of cancer [122]. Induced MCL1, an essential apoptotic regulator protein for the survival of stem and progenitor cells of multiple lineages, is expressed at abnormally high levels in B- and mantle-cell lymphomas, CML, and MM. While the mechanism of overexpression of MCL1 in cancer is not completely understood, USP9X is thought to stabilize MCL1 by removing degradative Lys-48–linked polyubiquitin chains. Increased expression of USP9X is highly correlated with increased MCL1 in diffuse B-cell lymphomas and MM. Knockdown of USP9X results in downregulation of MCL1, which enhances cell apoptosis in human follicular lymphomas and B-cell lymphomas [123]. Increased MCL1 and USP9X protein expression has been detected during relapses of AML, acute lymphocytic leukemia (ALL) [124] and MM [125], and is associated with increased tumor survival. Inhibition of USP9X by WP1130 downregulates MCL1 protein, inducing apoptosis in CML cell lines [103]. Selective silencing of USP9X in CML cell lines resulted in downregulation of MCL1 and increased sensitivity toward drug and apoptotic stimuli [126]. Preclinical trials with the USP9X inhibitors ABT-737 and ABT-263 demonstrated that they could increase proteasomal degradation of MCL1 through USP9X inhibition [123]. 

CML is associated with an abnormality in chromosomes, resulting in unregulated expression of Bcr-Abl, and causing aberrant tyrosine kinase activity. Bcr-Abl kinase inhibitors such as imatinib showed high efficacy in CML patients. However, long-term exposure to this drug results in acquired drug resistance and disease progression at later stages. In-depth analysis also showed that resistance to imatinib is correlated with an increase in expression of USP9X. Treatment with WP1130, an anti-leukemia drug, results in downregulation of Bcr-Abl and USP9X-mediated apoptosis in CML [126]. Another novel small molecule, EOAI3402143 (with properties similar to WP1130), selectively inhibits USP9X and USP24, induces apoptosis in malignant B-cell lines, and also blocks or regresses myeloma tumors in mice [127]. Inhibition or knockdown of USP9X may therefore be a therapeutic target in various hematological malignancies with abnormal USP9X activity.

USP9X also exhibits mitotic activity due to its role in the regulation of chromosome alignment and segregation by spindle assembly checkpoint (SAC) targeting survivin and Aurora B and other inhibitors of apoptosis proteins. SAC-induced mitotic arrest coupled with knockdown of USP9X are important targets for anti-neoplastic therapies [128]. USP9X binds specifically to numerous substrates in different types of cells. It also plays an important role in T-cell proliferation, T-helper-cell differentiation, and cytokine production [119,129]. Recent studies have demonstrated that USP9X deubiquitinates the X-linked inhibitor of apoptotic protein (XIAP) to promote mitotic survival in aggressive B-cell lymphomas through RNAi-mediated knockdown of USP9X. Overexpression of USP9X is also correlated with increased expression of XIAP, which has been identified as a predictive biomarker for chemotherapy resistance in diffuse B-cell lymphomas [129]. Indeed, USP9X is involved in the regulation of various mitotic and apoptotic proteins and its overexpression is associated with various hematological malignancies, making USP9X a potential theurapeutic target. Deeper insights into the mechanisms involved in signaling pathways associated with USP9X would help develop more effective drugs. In addition, unbiased determination of USP9X targets and its regulation may yield a more comprehensive assessment of DUB activity in cancer cells. Additional studies to determine key components in the apoptotic pathway and a role for USP9X in this process may help develop more effective cancer therapies. 

### 5.5. USP14

USP14 is a DUB associated with the 19S proteasome, which dynamically regulates the magnitude and nature of its activity, but its role in disease development is unclear [130]. Various studies have revealed that USP14 is associated with numerous types of cancer. Specifically, it was reported that upregulated expression of USP14 is associated with leukemia and may be implicated in apoptosis [131]. Various proteasome inhibitors, such as bortezomib [132], carfilzomib [133], and MLN9708 [134], have contributed significantly toward treatment and survival in MM and B-cell-related malignancies in patients. However, increasing development of resistance of cancer cells against chemotherapy has been a main roadblock in development of treatment for malignancies. Studies have shown that MM cells can be protected from chemotherapy-induced apoptosis by the phenomenon called cell adhesion mediated drug resistance (CAM-DR) [135]. USP14 reportedly contributes to CAM-DR by upregulating the anti-apoptotic protein Bcl-xl and Wnt 3 signaling pathways. Studies have shown that USP14 is significantly overexpressed in CAM-DR models and downregulated in apoptotic models of MM. Moreover, upregulation of USP14 in MM models could enhance anti-apoptotic cell-adhesion abilities, thus promoting drug resistance in MM [136]. 

Co-inhibition of USP14 and UCHL5 by novel DUB inhibitor VLX1570 revealed potent tumor-specific apoptotic activity in drug-resistant tumor cells of Waldenstrom macroglobulinemia (WM), an incurable non-Hodgkin lymphoma [115,137]. Recently it was also established that targeted inhibition of USP14 and UCHL5 with the novel small-molecule proteasome inhibitor b-AP15 induced proteotoxic stress and apoptosis in tumor cells of WM, without affecting proteolytic activity of the 20S proteasome [138]. The turnover of many cell-cycle regulatory proteins such cyclin-dependent kinase (CDK) 1A and 1B as well as p53 protein is controlled by b-AP15. Accumulation of cell-cycle inhibitors and regulatory proteins results in cell-cycle arrest, along with increased DNA damage markers [130], suggesting b-AP15 exhibits genotoxic properties. In addition, b-AP15–mediated inhibition of proteasome deubiquitinating activity suppressed tumor progression and organ infiltration in different in vivo solid tumor models of an AML [139]. b-AP15 mediated inhibition along with siRNA knockdown of USP14 and UCHL5 induces synergistic apoptotic activity in MM tumor cells and overcomes resistance to bortezomib [140]. A recent study involving selective inhibition of USP14 by IU1 treatment accelerated the degradation of proteins under proteotoxic stress in MM [141], but inhibition of both 19S proteasome-associated DUBs resulted in the accumulation of polyubiquitinated proteins [130]. Taken together, these results suggest a redundancy between USP14 and UCHL5, with either one required for proteasomal function. 

We propose that deubiquitinating activity of the 19S regulatory subunit of proteasome can be a potential pharmacological target for cancer treatment. Use of broad-spectrum DUB inhibitors along with proteasome inhibitors such as bortezomib can be effective in the treatment of hematological malignancies. Further investigation of the role of USP14 may provide a preliminary theoretical basis for its application in clinical research.

### 5.6. USP24

USP24 is a 2620-amino-acid protein containing a catalytic ubiquitin C-terminal hydrolase domain and an ubiquitin-associated domain. Although the function of USP24 is poorly understood, a recent study demonstrated overexpression of USP24 protein in certain cancer types during the later stages of disease progression. Studies have also demonstrated that USP24 promotes cancer malignancy by inducing IL-6 transcription into tumor-infiltrating leukocytes, vascular endothelial cells, and cancer-associated fibroblasts [142]. In another study, selective inhibition of USP9X by the inhibitor WP1130 reduced MCL1 levels and induced apoptosis in MM cells. However, significant upregulation of USP24 was observed when USP9X was inhibited, emphasizing its role in MM cell survival [143]. USP24 was also found to regulate survival of MM cells in absence of USP9X. Direct USP24 knockdown resulted in apoptosis of myeloma cells associated with a reduction in MCL1 levels. Dose-dependent inhibition of USP9X and USP24 activity by a modified compound of WP1130, EOAI3402143, increased cell apoptosis and completely regressed myeloma tumors in mice models [127]. Even though certain reports suggest an indirect role of USP24 in certain hematological malignancies, the function of USP24 in disease prognosis remains unclear. Further studies should help identify the role of USP24-mediated post-translational modification in the interaction of tumor cells and tumor associated microenvironment. 

### 5.7. CYLD

NF-κB transcriptional factors and associated signaling pathways play a central role in activation of the innate and adaptive immune responses, and are involved in cancer development, tumor angiogenesis, and progression [144]. CYLD is a negative regulator of NF-κB signaling and its loss inhibits apoptosis by activation and expression of NF-kB-dependent cells’ pro-survival genes [145]. The CYLD gene was first identified in association with suppression of multiple skin tumors in cases of familial cylindromatosis [146]. CYLD is an essential mediator of RIPK1- and RIPK3-dependent necroptosis [145].

Proteolytic cleavage of CYLD through the para-caspase MALT-1 tissue results in NF-κB activation, which is a key step in the initiation of T-cell acute lymphoblastic leukemia (T-ALL) [147,148]. Notch signaling regulates activation of the NF-κB signaling cascade and facilitates NF-κB nuclear retention during T-cell activation [149]. Dysregulated Notch gene expression is a common feature of acute T-ALL [150]. Recent evidence suggests that Notch-induced activation of NF-κB pathways plays a key role in T-cell leukemia, and the degree of downregulation of NF-κB is correlated with the severity of the disease [151]. CYLD-mediated suppression of NF-κB signaling and Iκ-B kinase (IκK) expression and function weakens human T-ALL cells and also represses tumor growth in animal models [152].

CYLD negatively regulates mitosis and cytokinesis [153,154], and plays an important role in the regulation of microtubule dynamics and cell migration [155,156] and apoptosis. CYLD can therefore be a novel target for the development of therapeutics against hematological malignancies. As is evident in previous studies, regulation of NF-κB signaling plays an important role in the initiation and pathogenesis of hematological malignancies. Along with CYLD, A20, and other DUBs, such as USP10 [157], USP11 [158], USP21 [159], USP15 [160], and OTULIN [161], play important roles in the activation or inhibition of the NF-κB pathway. The studies make it clear that DUBs play vital roles in ensuring optimal signal transduction and homeostasis through tight regulation of cell death and NF-κB activation. The identification of novel DUBs and cross-talk between DUBs, which may reveal further mechanisms and functions of these DUBs in the pathogenesis of hematological malignancies. Screening of DUB-conditional knockout cell lines or animal models will likely help identify novel DUB targets and clarify their functions beyond inflammation, tumor suppression, and immunity. Although the data available regarding the role of DUBs in hematological malignancies continues to grow, the ultimate challenge will be to apply and translate this fundamental knowledge to the development of effective and novel therapies. 

## 6. Deubiquitinases as Emerging Targets against Hematological Malignancies

Ubiquitination and deubiquitination play critical roles in various biological pathways closely associated with development of various cancers. Although knowledge about the precise role of DUBs in cancer pathology is limited, the list of DUBs that are altered genetically in human cancer cases has grown rapidly in recent years [162]. Recent studies have presented DUBs as a genuine oncogene and tumor suppressor. DUBs that regulate cellular expression and turnover of oncogenic protein in various hematological malignancies have been identified. In recent years, inhibitors of the UPS have emerged as therapeutic targets for the treatment of various cancers. However, application of most of these inhibitors are hampered by low efficacy in hematological malignancies [163]. The ability of DUBs to modulate the fate of a protein specifically and selectively gives them an advantage over targeting the UPS. For example, DUBs may increase or maintain levels of a tumor suppressor protein by decreasing its degradation by UPS or boost pathogenesis by reversing the fate of oncogenic proteins in the cell [26]. Considering the advantages and ease of developing inhibitors over enzyme activators, research into the development of DUB inhibitors against hematological malignancies has been emphasized.

Recent approaches to targeting DUBs through various small-molecule inhibitors have produced promising results against various hematological malignancies. The novel regulatory particle b-AP15 together with lenalidomide, or dexamethasone, induces synergistic anti-MM activity [140]. According to recent studies, b-AP15 and VLX1570 could also be a potential therapy for leukemia and WM by inhibiting 19S proteasome-associated DUBs such as USP14/UCHL5 and inducing tumor-cell apoptosis. VLX1570 along with dexamethasone was in clinical trial phase I/II but was terminated because of dose-limiting toxicity [164]. Another potent DUB inhibitor WP1130, previously known as Degrasyn, targets deubiquitinases such as USP5, USP9X, USP14, USP24, and UCHL5. Recent studies concluded that WP1130-mediated inhibition of USP9X increases ubiquitination of an anti-apoptotic protein MCL1 which is highly expressed in drug-resistant MM tumors [127,165]. The rapid degradation of MCL1 results in an increase in the sensitivity of these tumor cells to chemotherapy [26,163,166]. USP24, which is closely related to USP9X, also plays a critical role in the survival of myeloma B cells by regulating MCL1 protein levels. Peterson and colleagues suggested that dual inhibition of USP9X and USP24 by WP1130 provides greater anti-myeloma activity. However, they developed an inhibitor that is three times as effective called EOAI3402143 (G9), which also had an improved therapeutic index. G9 inhibits USP5, increasing p53 accumulation and therefore is a promising approach to treating B-cell malignancies [127,167]. At present, studies of USP7 and USP10 inhibitors (HBX19818 and P22077), which play key roles in many cellular processes, are under way. A lead-like inhibitor, HBX41108 and HBX19818, has been found to inhibit the catalytic activity of USP7 and induce p53-dependent apoptosis [168,169]. 

Preclinical data demonstrated the anti-tumor efficacy of P5091 with lenalidomide, histone deacetylase inhibitor SAHA, or dexamethasone by inducing tumor-cell apoptosis in MM disease models [118]. It also stabilizes p53 by inhibiting USP7 mediated-deubiquitination of MDM2, which degrades p53 tumor suppressors [115,170,171], thus inhibiting cancer cell proliferation. HBX19818, P5091, as well as their analogs, including P045204, P22077, and HBX41108 have been shown to be potent inhibitors of USP7 [172]. P217564, a second-generation inhibitor, binds to the active site of USP7, inhibiting its activity [173]. Along with USP7, P22077 and HBX19818 has also been reported to inhibit USP10, promoting degradation of FLT3-mutant AML cells [174]. A small-molecule inhibitor, spautin-1 (for *sp*ecific and potent *aut*ophagy *in*hibitor-1), inhibits autophagy, and two DUBs, USP10 and USP13, which deubiquitinates two tumor suppressors, Beclin1, a subunit of Vps34 complexes, and p53 [175].

Despite the development of several inhibitors against DUBs, none of them have gone on to clinical trials, likely due to the complex structure of the catalytic domain of DUBs that share similarities with other DUB family members. Upon ubiquitin-binding, the active sites of DUBs also undergo conformational changes, posing a challenge when designing as well as binding the inhibitor to its specific DUB. The UPS consists of two regulatory enzymes, E3 ligase, and DUBs, which work as a complex, and understanding the dynamics of the complex, not just the DUBs, is essential to engineering a specific inhibitor. Moreover, it has been well documented that diverse DUBs play crucial roles in many cellular processes. Future research should be channeled toward developing small-molecule inhibitors that target the conserved catalytic cysteine of DUBs with stable and selective substrate-binding efficacy using new technologies. A high-throughput screening method should be made available with which researchers can determine the combination of DUBs and/or inhibitors that best modulate active pathways in cancer.

A better understanding of regulatory DUBs involved in inhibition or activation of hematopoietic processes and pathologies is expected to open new frontiers in the development of novel therapeutic drugs that target hematological malignancies and disorders. The goals are to enhance our understanding of dysregulated DUBs in hematopoiesis; design new therapeutic targets, and establish biomarkers that could be used in diagnosis and prognosis.

## 7. Conclusions

Since its discovery, the UPS has emerged as a key regulator of various proteins and factors involved in hematopoiesis, erythropoiesis, and angiogenesis. The roles of E1, E2, and E3 enzymes in governing the various pathways involved in hematopoietic regulation and pathologies have been studied extensively, but knowledge about the reversal of the activity of DUBs and their involvement in various hematological processes is limited. Several studies provide insight into dysregulated functioning of DUBs in various hematopoietic cells, which contribute to hematological pathologies. In this review, we described various DUBs that directly or indirectly regulate various hematopoietic processes.

**Figure 2:**
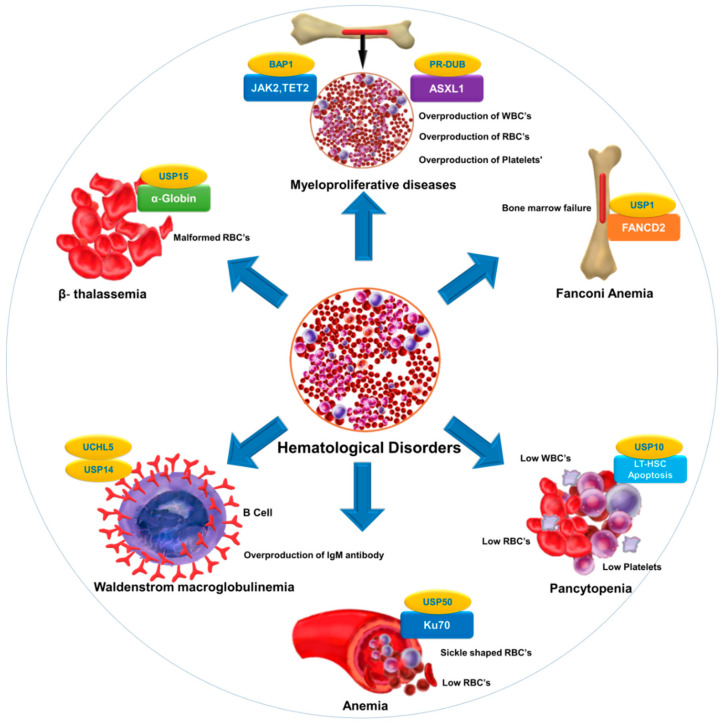
DUB as a novel target in hematological diseases. DUBs regulate the level and function of a protein by catalyzing removal of ubiquitin from the substrate protein. Dysregulation of DUBs contributes to the pathogenesis of various hematological disorders. The figure illustrates different hematological disorders and the associated DUBs that can provide novel targets for therapeutic interventions to treat these disorders.

Selective inhibition or overexpression of DUBs has helped elucidate their roles in hematopoiesis, erythropoiesis, angiogenesis, and related abnormalities. Potent selective inhibitors of DUBs have shown promise for the treatment of hematological malignancies. Hematological disorders are the result of one or more malfunctioning components in the blood caused by intrinsic factors. DUBs are thought to play an important role in the etiology of various diseases and disorders and are therefore attractive drug targets. However, limited knowledge about substrate specificity and the molecular mechanisms of action of DUBs currently restricts their utility as novel therapeutic targets (Figure 2). 

Table 1 provides a list of DUBs associated with blood disorders. Identification of other DUBs involved in the pathogenesis of other hematological disorders and more complete insight into the regulatory mechanisms of DUBs that govern disease progression will provide new perspectives in therapeutics.

Although DUBs may play important roles in the regulation of hematopoiesis, many questions about their involvement remain unexplored. The exact DUBs-mediated signaling pathways responsible for the progression of hematological disorders have yet to be elucidated. Much of this knowledge gap stems from the ability of a given DUB to regulate several hundreds of protein substrates. At the same time, a given protein substrate can be regulated by more than one DUB. 

Despite progress toward the development of DUB inhibitors, a lack of specificity limits clinical application of a wide range of DUBs [112]. This limitation may be overcome by improved understanding of self- versus trans-regulation of DUBs and applying this information to inhibitor synthesis prior to applying such DUB inhibitors to preclinical and clinical research. Furthermore, another possible mechanism of “dubbing” DUBs in cases where a particular DUB itself is regulated by another DUB should also be seriously considered [13]. Expanding research on dubbing DUBs is expected to offer novel insights into understanding DUB regulatory networks and implementing innovative strategies to uncover and develop molecular therapies to treat hematopoietic diseases. 

The DUBs discussed in this review raise the question of whether there is a master DUB regulator that can deubiquitinate multiple DUBs. However, conventional DUB-mediated therapeutic approaches involve targeting DUBs that contribute to hematological pathologies. Dubbing DUBs may allow for screening for a master DUB that regulates the key DUBs implicated in hematological pathologies. Once such master DUBs are identified, the task of generating selective and specific inhibitors of DUBs implicated in hematologic disorders may become easier. We hypothesize that mapping an exclusive inter-DUB regulatory network combined with wide proteomic-scale screening of crucial DUBs will increase our understanding of several unknown links that may be related to the DUB regulatory network in hematological malignancies. 

The role of the DUBs in HSC maintenance and differentiation remains an active area of research. Further investigation into the localization and substrate specificity of DUBs, their interactions with other hematopoietic factors, and other data gaps will improve our knowledge about their role in hematopoiesis. Research in this direction will facilitate the development of specific inhibitors against the key DUBs implicated in hematopoiesis. Doing so, while minimizing undesirable side effects, should lead to exciting new opportunities in treating hematologic malignancies.

## Figures and Tables

**Table 1:** List of DUBs involved in hematological disorders.

Disorder	Associated Substrate	Regulatory DUB	Reference
Fanconi anemia	FANCD2	USP1	[63,176]
Anemia	Ku70	USP50	[8]
β-thalassemia	α-globin	USP15	[177]
Pancytopenia	Reduction in LT-HSC	USP10	[31]
Myeloproliferative diseases	ASXL1, EZH2,JAK2,TET2	PR-DUB,BAP1	[178,179]
Waldenstrom macroglobulinemia (WM)	Overexpression of USP14 and UCHL5 in drug-resistant WM-tumor cells	USP14 and UCHL5	[137,180,181]
Bone marrow failure	B-cell factor 1 (Ebf1), paired box 5 (Pax5), and other B-lymphoid genes	MYSM1	[45,182]
Malaria	CD8+ T cells	CYLD	[162,183]

## Abbreviations:

AML	acute myeloid leukemia
BM	bone marrow
CAM-DR	Cell adhesion mediated drug resistance
CDK	cyclin-dependent kinases
CFU-E	colony forming unit erythroid
DDR	DNA damage response
DUBs	Deubiquitinating enzymes
FA	Fanconi anemia
FL	fetal liver
IL	Interleukin
LUBAC	Linear ubiquitin chain assembly complex
MALT-1	Mucosa-associated lymphoid tissue
MINDY	motif interacting with Ub-containing novel DUB family
MCPIP1	monocyte chemotactic protein–induced protein 1
MM	Multiple myeloma
NEMO	NF-kappa-B essential modulator
NK	natural killer
NF-kB	Nuclear factor-kB
OUT	Otu-domain ubiquitin aldehyde-binding proteins
PR-DUB	polycomb repressive deubiquitinase complex
PRC1	polycomb repressive complex 1
RBCs	red blood cells
SAC	spindle assembly checkpoint
TRAF	tumor necrosis factor receptor–associated factor
UPS	Ubiquitin proteasome system
USP	Ubiquitin-specific proteases
WM	Waldenstrom macroglobuliemia
XIAP	X-linked inhibitor of apoptotic protein
